# Relationship between quality of life and self-care ability in patients receiving hemodialysis

**Published:** 2010

**Authors:** Mehdi Heidarzadeh, Solmaz Atashpeikar, Tahereh Jalilazar

**Affiliations:** * School of Nursing, Instructor in Nursing, Bonab Branch, Islamic Azad University, Bonab, Iran; ** Expert Nurse, Member of Young Researchers Club, Bonab Branch, Bonab Islamic Azad University, Bonab, Iran

**Keywords:** Hemodialysis, quality of life, self-care

## Abstract

**BACKGROUND:**

Although hemodialysis has a therapeutic effect on end stage renal disease (ESRD), these patients encounter many physical, psychological, and social stressful factors that lead to a decrease in their quality of life (QOL). One of the factors that are effective on increasing the QOL is the self-care ability. Review of literature demonstrated a few studies done on different aspects of QOL in ESRD patients under hemodialysis and their relationships with self-care ability in Iran. So, in this research besides determining the quality of life and its dimensions and self-care ability of hemodialysis patients, we evaluated their relationships with each other.

**METHODS:**

For this purpose, all hemodialysis patients who had inclusion criteria and were hospitalized in hemodialysis wards of Bonab, Maragheh, and Miandoab hospitals were selected and data were collected by interview using a questionnaire that included three parts, demographic factors, quality of life, and self-care ability.

**RESULTS:**

The results indicated that quality of life in 34%, and self-care ability in 78.3% of hemodialysis patients were desirable, and there was a direct and significant relationship between these two variables (p < 0.001, r = 0.4), as self-care ability explained 29% of variance of QOL. In quality of life subsectors, social dimension in 98.3% of patients was desirable, while physical dimension (80%) and psychological dimension (63.5%) in most patients were undesirable. Physical dimension was the most impressible dimension of quality of life in self-care ability whereas self-care ability explained 27% of total variance of physical dimension of QOL.

**CONCLUSIONS:**

Nearly two thirds of mentioned patients had no desirable QOL and regarding the positive relationship between QOL and self-care ability, it is suggested that health care planner and managers prepare the condition that through educating and reinforcing self-care ability in these patients improve the QOL in hemodialysis patients.

Chronic Renal Failure (CRF) is a progressive destruction of kidney function in which the body metabolism and water and electrolyte balance would be disturbs resulting in uremia.^1^ The main treatment of end stage renal disease (ESRD) is kidney transplantation, but regarding the difficulties of kidney transplant, the patients should be treated with hemodialysis, until they find a kidney for transplantation. More than 60000 people die annually in the world because of renal failure.^2^ The incidence rate of chronic renal failure in the world is about 242 people from every 1’000’000, and it was increased 8% annually. The rate of incidence is variable in every country.^3^

Based on Iran’s Special Disease Centre and Kidney Disease supporting Association’s statistics, there are 267 dialysis centers in Iran (52 centers in Tehran, and 215 centers in other cities). There were about 20’134 progressive CRF patients until March 2004 that 10’276 of them were under hemodialysis. The number of patients that need hemodialysis is increasing 15% every year in Iran.^4^

Although hemodialysis has a therapeutic effect on ESRD, but these patients encounter many physical, psychological and social stress full factors that are not controllable even with new technologies.^5^ Lancaster said that the patients who treat whit hemodialysis would face many stressful factors in every aspect of their life such as family problems, change in sexual function, become dependent to others for surviving, social isolation, changing in body image, mental stresses, and suicide.^6^ Rittman reported that in CRF patients, uremic signs of ERSD, non kidney physical disorders, daily activity problems, and the problems that drive from therapeutic ways are the main problems in these patients.^7^ The patients who receive hemodialysis encounter many pathological states such as hypertension, lack of appetite, anemia, genital disorder such as change in menstruation, skin disorders such as itching, change in skin color and arteriovenous fistula.^8^

In the recent years, quality of life (QOL) is one of the concepts that have been accepted as a criterion for evaluating the result of medical efforts, and situation of patients with mental and somatic disorders.^9^ Testa et al (1998) defined different effective dimensions of quality of life as following; physical, psychological, and social dimensions of health that are affected by individual’s experiences, beliefs, expectations, and perceptions.^10^ So, QOL should be measured regarding different dimensions including physical, psychological, and social ones.^11^ Physical and psychological disorders have an important role in the decrease of quality of life in hemodialysis patients.^2^ Different factors can be effective in increasing QOL in hemodialysis patients. One of them is self-care ability. Regard to Orem’s theory, self-care is a learnable behavior that would solve client’s general, developmental and health deviation needs.^12^ Orem wrote that self-care ability is the continuous efforts that people do themselves to continue their life, and to provide health and welfare. Adult people have this ability, but newborns, infants, old people, patients, and disable people in doing self-care, are either completely dependent or needful to others.^12^ The studies showed that there is a positive relationship between QOL and self-care ability. Tsay et al showed that self-care ability and depression have significant effect on QOL, as self-care ability explains 47.5% of variance of QOL.^13^ Other studies also showed a positive relationship between self-care ability and different aspects of QOL; for example Masoudi et al showed that self-care education programs can improve physical aspect of quality of life in multiple sclerosis patients.^14^ In another study Madani showed that self-care programs can increase self-esteem, improve muscular contraction, relive tiredness, improve constipation, reinforce memory, and declare amnesia in multiple sclerosis patients.^15^ Other studies also showed that self-care programs can improve quality of life in patients with leukemia, liver cirrhosis, and multiple sclerosis.^16^–^18^

Few studies have been done about the quality of life especially in the field of self-care ability in hemodialysis patients in Iran community. Also limited works have been done about different dimensions of QOL, and relationship between these variables, and whereas one of the nursing goals is improving of self-care ability in patients until they acquire their independencies, and prevent many complications that treated them.^19^ The aim of this study was to determine the quality of life and its different dimensions in hemodialysis patients and to detect self-care ability in hemodialysis patients and correlation between quality of life and self-care ability in hemodialysis patients.

## Methods

This was a cross sectional study. This study has been done in 2008. Target population of this study was all of the patients who were receiving hemodialysis in dialysis wards in Imam Khomeini hospital in Bonab city, Sina hospital in Maragheh city, and Abbasi hospital in Miandoab city. After sample selection, they were interviewed to evaluate their self-care ability and QOL using a prepared questionnaire. Regarding the small number of patients in these wards, all of qualified patients that were satisfied to participate in this study were selected as sample after explaining the study and assuring them about confidante of study. The inclusion criteria were as following; they should received hemodialysis 2-3 times a week; they should have been started treatment with hemodialysis at least since 3 months ago^20^; satisfaction to participate in this study; they could write or communicate; and lack of history of sever psychological disorders such as schizophrenia and other psychological diseases that we can’t trust the patient’s states.

### 

#### Instrument

Quality of life questionnaire includes 3 parts; 1. physical dimension (12 items), psychological dimension (10 items), and social dimension (12 items). This questionnaire has been composed of two questionnaires: SF36 quality of life questionnaire^13^; and Sweden health related quality of life questionnaire.^21^ Self-care ability was measured by the questionnaire that was provided by the researcher by using instruments of previous researches and literatures.^22^ These questionnaires were used after becoming valid (content validity), and reliable. Alpha Coronbach coefficient was used for determining reliability, and content validity for determining validity. Alpha Cronbach coefficient result the following for every item of questionnaires; Physical function (0.93), pain and ailment (0.81), sexual activity (0.82), sleep (0.77), psychological dimension of quality of life (0.74); social dimension of quality of life (0.79) and for determining reliability of self-care ability, in general caring dimension Alpha Cronbach coefficient was 0.78, and for other questions Kuder Richardson was used that it was significant. Then collected data was analyzed by SPSS (ver. 15) using X^2^, Pearson coefficient, multiple regression analysis. P-value under 0.05 was considered to be significant.

## Results

Most of the patients (50.5%) were 39-62 years old; the mean age (SD) was 50.2 (15.4); 51% were male, 77.4% were married, 58% were un-employed, 54.8% were illiterate, 59.3% of patients had received hemodialysis for 1-5 years, and 77.4% of them were receiving hemodialysis 3 times every week; 91.2% haven’t been transplanted before.

The study showed that 66% of patients had undesirable quality of life (mean = 131.3, SD = 21.1), furthermore 80% had undesirable quality of life regarding physical dimension and 63.5% regarding psychological dimension while 98.3% had desirable quality of life in social dimension (Table 1).

In self-care ability, the results showed that 78.3% of patients had desirable self-care ability. The most desirable self-care ability in patients was related to “care of arteriovenous line” and the least of them was related to nutrition field (Table 2).

**Table 1:** Quality of life and its dimensions in hemodialysis patients

Variable		Desirable- Undesirable	Frequency	Percent	Mean	Standard deviation
		Undesirable (134.5 - 221)*	76	66		
Total Quality of life		Desirable (48 - 134.5)	39	34		
		Total	115	100	131.3	21.1
		Undesirable (> 72)	92	80		
	Physical subsector	Desirable (> 72)	23	20		
		Total	115	100	59	15.3
		Undesirable (< 29.5)	73	63.5		
Dimension of Quality of life	Psychological subsector	Desirable (> 29.5)	42	36.5		
		Total	115	100	28.9	5.7
		Undesirable (< 33)	2	1.7		
	Social subsector	Desirable (> 33)	113	98.3		
		Total	115	100	43.3	4.4

* The number in the parenthesis is the summation of the scores that the patients gave in the instrument. These scores are divided into two parts regarding the median.

**Table 2:** Self-care ability in hemodialysis patients

Variable		Desirable- Undesirable	Frequency	Percent	Mean	Standard deviation
		Undesirable (5 - 30.5)*	25	21.7		
Self-care ability		Desirable (30.5 - 56)	90	78.3	35.7	6.3
		Total	115	100		
		Undesirable (0 - 6.5)	31	27		
	Care of vessel line	Desirable (6.6 - 13)	54	73	7.8	2.2
		Total	115	100		
		Undesirable (0 - 11)	39	33.9		
Dimension of self-care ability	Dietary	Desirable (11.1 - 22)	76	66.1	12.7	2.6
		Total	115	100		
		Undesirable (5 - 13)	38	33		
	General caring	Desirable (13.1 - 21)	77	67	15.2	3.8
		Total	115	100		

* The number in the parenthesis is the summation of the scores that the patients gave in the instrument. These scores are divided two parts regard to median.

The study showed that there was a direct and significant relationship between quality of life and self-care ability (p < 0.05), as self-care ability explained 29% of variance of QOL. There were direct and significant relationships between self-care ability and physical, psychological, and social dimensions of QOL too (p<0.05). It was notable that Physical subsector, was the most impressible aspect of QOL from self-care ability, so that self-care ability explained 27% of total variance of physical dimension of QOL, but it explained 17% of total variance of psychological dimension of QOL, and about social dimension it was only 5% (Table 3).

**Table 3:** The most impressible dimension of quality of life from self-care ability in hemodialysis patients

P	R^2^	Beta	Independent Variable	Dependent Variable	
<0.001	0.27	0.52	Self care ability	Physical subsector	
<0.001	0.17	0.42		Psychological subsector	Quality of life
0.01	0.05	0.23		Social subsector	
<0.001	0.29	0.54		Total	

Multiple regression analysis showed that self-care ability explained 29% of total variance of quality of life. Physical dimension was the most impressible dimension of quality of life from self-care ability.

## Discussion

Our result showed that more than half of the patients had undesirable quality of life. Tsay and Marilyn reported that hemodialysis patients encounter many physical, psychological, and social problems that result in decreasing of QOL in these patients.^13^ Evans et al performed a study on patients in a dialysis and kidney transplantation center; they showed that only 47.5% of hemodialysis patients had normal daily function.^23^ Also in another study in Taiwan, it was demonstrated that quality of life index in patients receiving hemodialysis was less than patients with kidney transplantation, breast cancer, colon cancer, and leukemia.^13^

The result showed that hemodialysis patients had few problems in social dimension, but they had more problems in physical and psychological dimensions of QOL. It supports previous studies. DeOreo showed similarly that the most undesirable dimension of QOL was physical one.^24^ Also, Mapes said that psychological problems explained 21% of the total variance of quality of life in hemodialysis patients.^25^ As mentioned above, hemodialysis patients have undesirable quality of life in physical and psychological dimension, but they have desirable quality of life in social one. It is due to good and suitable communication between these patients and their families, and the effective and durable support of family.^26^ Another study by Heidarzade et al showed that social support was desirable in most of the hemodialysis patients in Iran.^27^

The result of our study showed that two thirds of hemodialysis patients had desirable self-care ability. More than half of the patients acquired desirable score in self-care ability’s subsectors (caring of vessel line, observing diet, and general self-care ability). In the Tsay’ study, hemodialysis patients, acquired moderate score too.^13^

Self-care ability is compliance and power of the individuals in compiling the needs of the self-care that it has named “agency” in Orem’s self-care pattern. Orem said that adult people have self-care ability, but newborns, infants, old people, patients, and unable people in doing self-care, are either completely dependent or needful to others.^12^

Also our results showed that there was a direct and significant relationship between quality of life and self-care ability. Also, other studies showed that there was a positive relationship between quality of life and self-care ability. Leo and Owen found a significant relationship between QOL and self-care ability in 97 hemodialysis patients.^13^ Pardanjani et al showed that a self-care education program in hemodialysis patients can increase QOL in them.^8^ It seems that the patients with better self-care ability can observe diet and care the vessel line and other requirements better, so they can control many physiological complications. It results in improving physical, psychological and social situation. Therefore it causes the improvement of the total Quality of life.

There were direct and significant relationships between self-care ability and physical, psychological, and social dimensions of QOL too. It was inferred that Physical subsector, was the most impressible dimension of QOL from self-care ability. Although hemodialysis leads to the decrease of symptoms and signs of renal failure, but it can’t change the process of disease and supersede the kidney completely, so patients encounter many problems such as hypertension, lack of appetite, anemia, decentralization for a long time, renal steodystrophia, genital disorder such as change in period of mensturation, skin disorder such as itching, and infection of arteriovenous fistula.^8^ The patients with high self-care ability can observe suitable diet and can take care of arterivenous fistula well, so they can prevent many complications that threaten their life. Therefore we can expect that physical dimension of QOL and self-care ability has a strong correlation with each other.

Although hemodialysis has a therapeutic effect on ESRD, but these patients encounter many physical, psychological, and social stress full factors that lead to the decrease of their quality of life. This study demonstrated that there are positive and significant relationship between quality of life and self-care ability; and whereas one of the nursing goals is improving the self-care ability in patients until they acquire their independency, and prevent many complications that treated them^19^; so, besides the emphasis on residual abilities, we should help the patients to earn their independency in self-care^28^ ; so, responsible people and nurses can increase self-care ability (through decreasing many physiological complications by doing some interventions such as determining self-care needs in patients; determining precedence of the problems; determining short-time and long time objects for solving the problems; training and giving self-care program in different theme such as dietary, care of arteriovenous line) until prepared the condition that quality of life will be increased.

The authors declare no conflict of interest in this study. Ethical committee approved the study.
